# A rare sequela of snake bite: latent calcific myonecrosis of the tibialis anterior

**DOI:** 10.11604/pamj.2026.53.158.51991

**Published:** 2026-04-14

**Authors:** Najeebuddin Patel, Priyanka Nagaonkar

**Affiliations:** 1 D.Y. Patil Medical College, Hospital and Research Centre, Pune, India

**Keywords:** Calcific myonecrosis, snake bite, myositis ossificans

## Image in medicine

A 50-year-old male presented with a long-standing, slowly progressive, hard swelling over the anterolateral aspect of the right leg. He reported a history of a snake bite to the same limb 20 years earlier, which was associated with severe local tissue injury. There was no history of recent trauma, constitutional symptoms, or clinical signs of active infection. A plain radiograph of the right leg demonstrated sheet-like intramuscular calcification along the lateral aspect of the tibia without cortical erosion or periosteal reaction. Non-contrast computed tomography (CT) revealed well-defined linear intramuscular calcifications with central areas of low attenuation suggestive of necrosis within the anterior compartment of the right leg. Magnetic Resonance Imaging (MRI) of the right leg demonstrated linear sheet-like intramuscular areas of T1- and T2-hypointensity corresponding to calcifications, with heterogeneous central signal intensity involving the tibialis anterior muscle. The differential diagnosis of such radiological features in the lower limb includes myositis ossificans, soft tissue sarcomas, and chronic expanding hematoma. Myositis ossificans classically demonstrates a zonal pattern of peripheral mature ossification with central lucency on radiographs or CT, which is not identified in this lesion, as there is no organized cortical bone formation. Soft tissue sarcoma may present with irregular or amorphous calcifications, a solid enhancing soft tissue component, and occasionally cortical erosion or periosteal reaction-features that are not observed in our case. A chronic expanding hematoma can show a well-defined capsule, internal fluid-fluid levels, and a hemosiderin rim on MRI. However, the plaque-like peripheral dystrophic calcification along a single muscle compartment is more typical of calcific myonecrosis. The absence of aggressive characteristics and the presence of fusiform compartmental involvement with sheet-like calcification support the diagnosis. Taken together, the imaging findings are most consistent with calcific myonecrosis rather than the above differentials.

**Figure 1:**
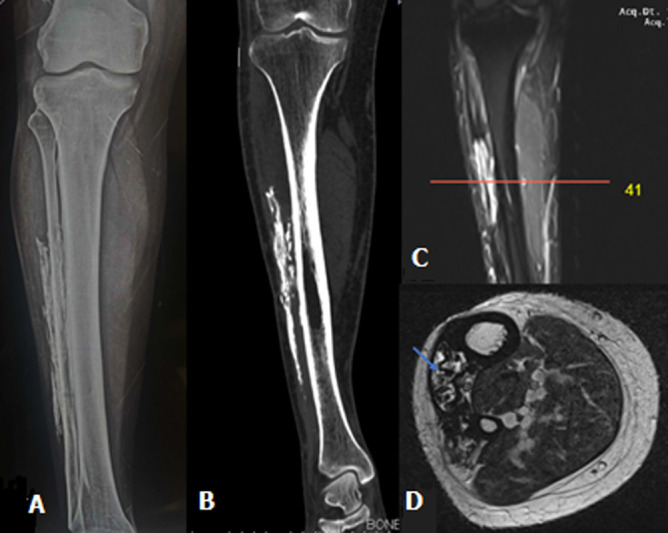
A) X-ray of the right leg demonstrating linear, sheet like, eggshell calcification in the soft tissue compartment laterally adjacent to the fibula in the mid-leg region showing no evidence of bone involvement or periosteal reaction; B) CT of the right leg coronal view, demonstrating peripheral sheet-like calcification along the long axis of the limb within a fusiform intramuscular lesion; C,D) MRI of the right leg (coronal and axial views) showing a fusiform intramuscular lesion with a low-signal peripheral rim and central T2 hyperintense calcified content (blue arrow)

